# Unfolded deep kernel estimation-attention UNet-based retinal image segmentation

**DOI:** 10.1038/s41598-023-48039-y

**Published:** 2023-11-24

**Authors:** K. Radha, Karuna Yepuganti, Saladi Saritha, Chinmayee Kamireddy, Durga Prasad Bavirisetti

**Affiliations:** 1 School of Electronics Engineering, Vellore Institute of Technology, Vellore, India; 2 School of Electronics Engineering, VIT-AP University, Amaravathi, Andhra Pradesh India; 3 Department of Computer Science, Norwegian, University of Science and Technology, Trondheim, Norway

**Keywords:** Engineering, Computational biology and bioinformatics, High-throughput screening

## Abstract

Retinal vessel segmentation is a critical process in the automated inquiry of fundus images to screen and diagnose diabetic retinopathy. It is a widespread complication of diabetes that causes sudden vision loss. Automated retinal vessel segmentation can help to detect these changes more accurately and quickly than manual evaluation by an ophthalmologist. The proposed approach aims to precisely segregate blood vessels in retinal images while shortening the complication and computational value of the segmentation procedure. This can help to improve the accuracy and reliability of retinal image analysis and assist in diagnosing various eye diseases. Attention U-Net is an essential architecture in retinal image segmentation in diabetic retinopathy that obtained promising results in improving the segmentation accuracy especially in the situation where the training data and ground truth are limited. This approach involves U-Net with an attention mechanism to mainly focus on applicable regions of the input image along with the unfolded deep kernel estimation (UDKE) method to enhance the effective performance of semantic segmentation models. Extensive experiments were carried out on STARE, DRIVE, and CHASE_DB datasets, and the proposed method achieved good performance compared to existing methods.

## Introduction

Retinal vessel segmentation in diabetic retinopathy is a key indicator of disease progression and treatment response for identifying and quantifying various features related to the eye’s vascular system. These features include vessel size and shape, microaneurysms, exudates, hemorrhages, and the degree of vascular occlusion. Clinicians and researchers can perform a detailed analysis of these features and monitor changes over time by segmenting the retinal image into distinct regions of interest. Hemorrhages and microaneurysms are initial clinical signals of DR (diabetic retinopathy). These are shown as small red dots and blotches in the retinal image. Segmentation of retinal vessels can assist in identifying and quantifying the lesions, which are important indicators of DR. The growth of new retinal vessels indicates proliferative DR. Segmentation of the retinal vasculature can assist in detecting and localizing these abnormal vessels, which can lead to complications such as vitreous hemorrhage and retinal detachment. DR can generate changes in the vasculature of the retina, such as narrowing of vessels, tortuosity, and increased vascular permeability. Segmentation of the retinal vessels can allow for the quantification of these changes, which can be used to monitor disease progression and response to treatment. The severity of DR is classified into different stages based on the existence and extent of lesions in the retina. Segmentation of the retinal vasculature can assist in the accurate classification of DR and in the identification of patients at higher risk for vision loss.

Automated retinal segmentation using machine learning algorithms has been shown to be effective in detecting and quantifying various features associated with diabetic retinopathy. It is a reliable approach to increase the accuracy and efficiency of this process. For example, DL (deep learning) applications with CNNs (convolutional neural networks) can be trained to segment retinal images and detect the presence of microaneurysms and other lesions associated with diabetic retinopathy. This can help clinicians identify early signs of the disease and monitor its progression over time.

**Figure 1:**
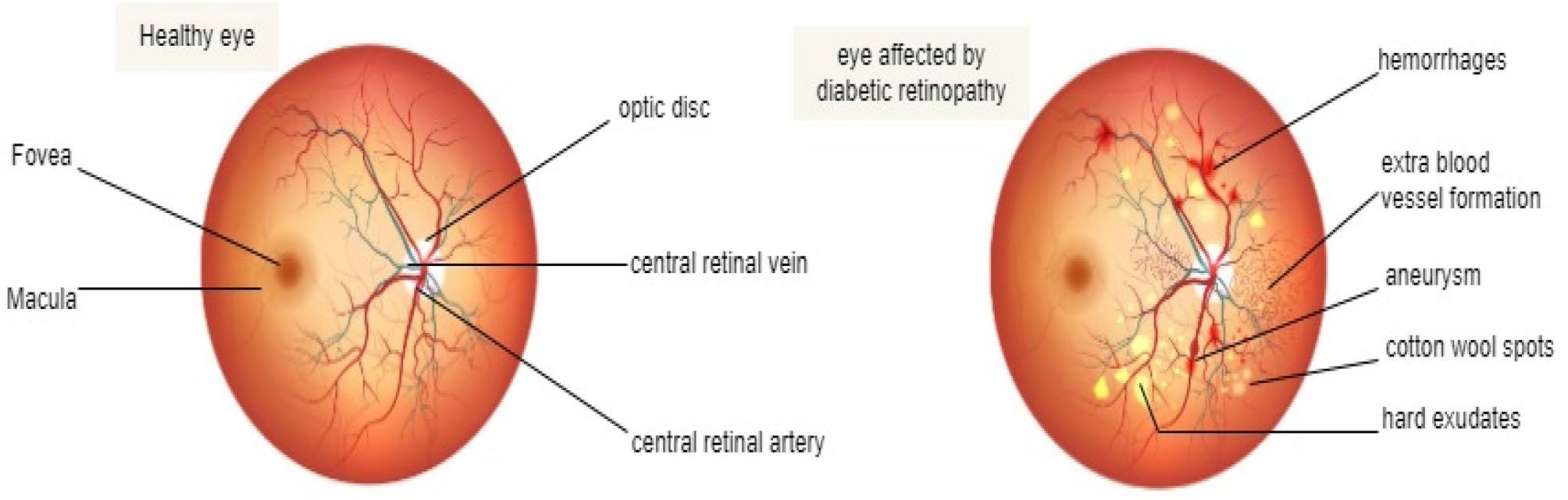
Difference between a normal eye and an eye affected by diabetic retinopathy.

CNNs are widely used in retinal vessel segmentation due to their skill in learning automatically to extract features in retinal images. CNNs are a deep learning algorithm consisting of multiple layers of interconnected nodes designed to identify patterns and features in the input image. By learning from large annotated data sets, CNNs can be trained to recognize and segment the vessels in retinal images. By training the network to recognize patterns of pixels that correspond to vessels, the network can then accurately segment new images, even in the existence of noise, low contrast, and few image artifacts. CNN is thoroughly designed to learn hierarchical features from input data, particularly useful for retinal vessel segmentation. Retinal images have a complex structure, and the vessel network is composed of varying widths, curvatures, and lengths showed in Fig. 1. CNNs can also capture these complex structures by learning hierarchical features that represent the appearance of different vessel components, such as the vessel walls, bifurcations, and crossings. Retinal images can vary in contrast, illumination, and resolution. CNNs are robust to these variations because they can learn features invariant to changes in image appearance. This means CNNs can still perform well even when retinal images have low contrast, poor illumination, or low resolution. CNNs have shown high accuracy in retinal vessel segmentation, outperforming traditional image processing techniques such as edge detection and thresholding. CNNs can accurately segment retinal vessels and detect fine vessel details such as thin vessels and branching points.

A DL architecture can be used for automated segmentation of retina vessels in fundus images, including multiple levels of CNNs with skip connections to extract features at different scales. To judge the model's performance in terms of sensitivity, accuracy, and specificity, outperforming other existing methods, the multilevel deep supervised network is shown to be sturdy in differentiating variations in image quality and lighting conditions^1^. For automated recognition of diseases in retinal fundus images, the optimal color channel that compares the performance of red, green, and blue color channels in detecting various pathologies in the retina, including DR, macular degeneration due to age, and glaucoma can be used. A DL-based approach using CNNs is helpful to pull out features from different color channels and classify retinal pathologies. Analysis indicates that the features taken out by CNN found that the green channel better captures the subtle variations in retinal structures corresponding with different pathologies^2^. To pull out features from input images, a CNN-based approach can be utilized in which features can be further processed by an attention mechanism to strengthen the informative features. This attention mechanism dynamically weights the feature maps established based on their significance in the segmentation task. This approach may sometimes be sensitive to image acquisition and quality variations, which can affect the segmentation performance^3^. To convert feature maps of the CNN model into a feature vector with a fixed length, the GAP (global average pooling) technique can be used, which is further fed into a fully connected layer for classification. This is similar to the identification of medicinal plants using deep CNNs that use leaf image datasets to train a deep CNN model that has the ability to accurately classify plant species^4^. To incorporate a mix of both high-level and low-level feature maps to increase the segmentation performance, a deep learning-based approach for segmenting blood vessels in retinal images named the multilevel attention network (MLAN) is useful. The MLAN has a decoder and an encoder architecture consisting of skip connections and several convolutional and pooling layers. The attention mechanism is applied at multiple levels of the encoder and decoder to strengthen the informative features and suppress the irrelevant features^5^.

Network inserted with skip connections allows the incorporation of both local and global context information between a decoder and an encoder in the UNet architecture allowing for the transfer of feature maps of high resolution from the encoder to the decoder, improving segmentation accuracy. The U-Net’s encoder part of the architecture contains max-pooling and convolutional layers capturing multiscale features of input images, and the decoder path includes upsampling along with convolutional layers that retrieve the spatial resolution of output segmentation maps. This architecture has become a widely used popular model for biomedical image segmentation, as well as other image segmentation tasks. However, significant amounts of annotated training data may be required to achieve optimal performance, which may be difficult to obtain for certain biomedical applications^6^. Incorporating recurrent connections into the decoder can help to predict long-range dependencies. Skip connections can also be utilized to connect respective layers from the encoder to the decoder pathway to transfer high-resolution feature maps and improve segmentation accuracy with RCSU-Net (recurrent convolutional skip connection U-Net). This network includes encoder and decoder networks. Convolutional layers and max pooling layers in the encoder pathway, along with convolutional layers and upsampling in the decoder. However, it requires a large amount of computational resources to use in real-time applications^7^. To replace the traditional recurrent and convolutional neural networks that use self-attention mechanisms that allow the model to contemplate the significance of different input and output elements depending on the context of the sequence, a transformer-based model is helpful. The Transformer architecture has an encoder and a decoder, both using multiheaded self-attention mechanisms to address different parts of the output and input sequences. Additionally, using residual connections and layer normalization helps with training and prevents overfitting^8^.

Using both pixel-level and feature-level encoding to capture multiscale context data with a dual encoding U-Net can improve segmentation accuracy. The dual encoding U-Net consists of two encoding branches, one that encodes the input image at the pixel level using convolutional and max-pooling layers and the other that encodes the feature-level information using dilated convolutional layers. The two encoding branches are then merged using skip connections to produce the final segmentation map. However, it requires a large number of computational resources, making it exigent to utilize in real-time approaches or perhaps on low-end devices^9^. Therefore, to enhance the performance of the model, a modified version can be proposed, for example, a deep UNet model that uses a dataset of retinal images in training that can accurately segment the vascular and nonvascular structures. The pruning technique can be used to remove redundant and insignificant features from the model, which can enhance the model’s efficiency and reduce overfitting^10^. To selectively highlight informative channels and reduce noisy channels in CNNs, work such as the ECA (efficient channel attention) module can be provided. Utilizing global knowledge from each channel, the ECA module computes channel-wise attention mappings via a straightforward 1D convolution technique. The result feature maps of the preceding convolutional layer are then weighted using the attention maps, increasing crucial features and decreasing unnecessary ones. Nevertheless, it only considers channel-wise attention and ignores spatial relationships between various portions of the image. This restriction could result in less-than-ideal performance on tasks requiring spatial information, including supervised classification or object recognition^11^. Another specification is to dissect the input image into several zones of different sizes and scales to extract features from each region using max-pooling, and a pyramid pooling module is helpful. The extracted features are concatenated and inserted into a thoroughly connected layer to assemble a global feature vector representing the image's overall context. This context vector is combined with feature maps generated by a CNN to generate more robust segmentation results. This pyramid pooling module helps capture fine-grained details and coarse-scale context information from the input image, which are essential for accurately segmenting complex scenes. Still, it only captures context information in a fixed set of scales, which may limit its ability to handle images with varying scales or aspect ratios^12^.

Additionally, for segmenting curvilinear features such as blood vessels and nerves in medical images, a new CNN called CS2-Net can be introduced, which uses attention mechanisms and dilated convolutional layers. S2-Net utilizes dilated convolutional layers to increase the network’s receptive field, allowing it to encapsulate more global context data from the input image. The network also incorporates attention mechanisms that selectively focus on the curvilinear structures of interest, enhancing their visibility and suppressing noise. However, the input image quality may affect the network's performance, such as low resolution or low contrast, which may require additional preprocessing steps to improve segmentation accuracy^13^. A coarse-scale CNN that executes the entire input image and a series of fine-scale CNNs can be used to stay focused on smaller zones of the retinal image. This is called MSFNet (multiscale network followed network), which uses multiple scales of CNNs. The fine-scale CNNs are trained to identify the blood vessels and generate probability maps, which are then merged using a fusion module to produce the final segmentation map. It can handle images with varying illumination and noise levels, making it a robust tool for clinical applications; however, a detailed description of the preprocessing methods used to prepare the retinal images for segmentation is not provided, which may limit reproducibility^14^. To distinguish between vessels and nonvessels, a 2D Gabor wavelet can be used, and a supervised classification algorithm can be used to extract vessel features. This method encapsulates the consistency and shape of features of retinal vessel images. The wavelet response is then thresholded to obtain binary vessel maps. The binary maps are further utilized as input of the supervised classification algorithm to categorize each as a vessel pixel or a non-vessel pixel. This classification algorithm is trained on a collection of labeled retinal images that uses a collection of features extricated from binary vessel maps to classify each pixel. However, this method relies heavily on the quality of Gabor wavelet parameters and the thresholding process, which may require significant tuning for optimal performance^15^.

To obtain appropriate results in any method, fundus image segmentation is considered the basic significant step for further processing. Therefore, a deep learning-based method should be introduced for segmenting vessels in retinal fundus images. One of them is the deformable convolutional neural network (DCNN). This method, also called DUNet, utilizes deformable convolutions to capture retinal vessels’ spatial variability and shape. However, this method may require massive computational resources and training data to perform well^16^. Another such method for medical image segmentation is using a recurrent residual U-Net (R2U-Net) architecture. This method incorporates recurrent connections in the input data to encapsulate long-range dependencies and residual connections to improve the flow of gradients during training. However, this method is limited to 2D image segmentation and may not directly apply to 3D image segmentation tasks^17^. To alleviate the vanishing gradient problem, a method similar to that used for road extraction in satellite images can be used for retinal vessel segmentation. This comprises a U-Net encoder-decoder architecture with residual connections that help increase the overall model performance^18^. For segmenting retinal blood vessels, a three-stage DL-based model can be used. This method consists of a coarse segmentation stage using a CNN, a proper segmentation stage using a dilated CNN, and a postprocessing stage using morphological operations. However, this method may not establish proper images received from different devices^19^. Furthermore, a trainable filter-based approach for vessel delineation in retinal images can be utilized to detect complex image features such as vessel junctions and crossings. The method combines trainable COSFIRE (combination of shifted filter responses) filters. However, this may not work well in complex images with overlapping vessels or noisy backgrounds^20^.

A method called brain-inspired wavelet transforms and random forest classification can be introduced for segmenting vessels in retinal fundus images. However, this method may not derive images well obtained with different imaging settings^21^. A method for retinal vessel segmentation that combines unsupervised feature learning and visual attention mechanisms can be utilized. This method uses a stacked denoising autoencoder (SDAE) to learn robust features for vessel segmentation and a visual attention mechanism to concentrate on the most factual regions of the image. However, the method’s interpretability and explainability may be limited due to unsupervised feature learning and visual attention mechanisms, making it difficult to understand how the method makes its predictions or to identify sources of errors and artifacts^22^. To enhance vessel segmentation in retinal fundus images, a method for segmentation that utilizes a cross-modality learning approach can be utilized, where features learned from optical coherence tomography (OCT) images are used. This method has three stages: OCT feature extraction, cross-modality feature transfer, and vessel segmentation. However, this may require OCT images to be acquired alongside fundus images, limiting its applicability in clinical practice^23^. Some existing methods may not achieve high accuracy in detecting retinal vessels, especially when dealing with low-contrast images, image artifacts, and noise, leading to significant computational resources and time requirements, making them impractical for real-time applications. Most existing methods are designed and optimized for a specific dataset, which may limit their generalizability to other datasets and applications needing manual parameter tuning, which can be time-consuming and challenging to perform optimally. Some of the current methods lack interpretability and transparency, making it difficult to understand how they work and how they make decisions. This may lead to failure in detecting retinal vessels in the presence of pathologies, such as exudates, drusen, and hemorrhages. These limitations suggest the need for improvements in the active field of retinal vessel segmentation to develop more accurate, efficient, and reliable methods.

To extract features of retinal images, the UNet architecture is introduced in this method, as discussed in section III-A, since it allows for accurate segmentation of the complex and fine-grained vessel structures in retinal fundus images. Furthermore, to increase the performance of the UNet architecture, the dropout block is modified in the original UNet architecture detailed in section III-B, which introduces a dropout and a dense layer in place of each convolutional block. An atrous channel attention block is inserted into the UNet architecture, as discussed in section III-C, to improve the capacity of the network and to represent subtle intricate details in the retinal vessels, which initiates a channel attention mechanism that selectively amplifies important features in each channel of the feature map. To allow an accurate segmentation of complex structures in retinal images, unfolded deep kernel estimation (UDKE) is introduced as discussed in section III-D, which combines deep neural networks with the flexibility of kernel-based methods. UDKE works by learning a set of convolutional kernels that can be used to estimate the pixel-level likelihood of each class in the segmentation map. The kernel-based approach allows for efficient estimation of the pixel-level likelihoods. At the same time, the deep neural network architecture provides a powerful feature extractor that can learn meaningful representations from the input image. To incorporate contextual information from multiple scales of the image, the multiheaded attention technique, as in section III-E, involves using a series of attention mechanisms to selectively highlight instructive features at consecutive scales of input images, which can boost the ability to record both global and local vessel structures of the network. This attention model is particularly effective for capturing vessel structures that vary in size and shape, such as small capillaries and larger vessels. By selectively attending to different image scales, the network can capture vessel structures that might be missed or misclassified by a single-scale approach.

The contributions of this paper are summarized as follows,

1. We have used UNet as a base network for the retinal vessel segmentation since we have limited ground truth for training the network.
2. The UDKE module is used in the convolution layer to learn important features from fundus images. Unfolded deep kernel estimation helps identify complex patterns and features, especially in the downsampling path. This enhances the network’s ability to learn relevant features for retinal vessels and distinguish them from other structures.

A preprocessing technique named CLAHE (contrast limited adaptive histogram equalization) is described in section IV-A, which can be utilized to supplement the contradiction of the retinal image. In CLAHE, the contrast of a retinal image is enhanced by dividing the image into small subregions, calculating the histogram of each subregion, and redistributing the pixel values based on the cumulative distribution function of the histogram. By limiting the contrast enhancement to each subregion, CLAHE avoids overamplifying noise and other image artifacts that can negatively affect the segmentation performance. CLAHE has been shown to be particularly effective for enhancing the contrast of low-contrast retinal images, which can improve the propensity of the segmentation algorithm to differentiate and determine a vessel pixel from a non-vessel pixel. By improving the contrast of the retinal image, CLAHE can help to reduce the influence of the image artifact and variation. After proper training and testing, as in section IV-B, these results are evaluated in detail by a few evaluated metrics discussed in section IV-C.

## Terminology

### Diabetic retinopathy

Diabetic retinopathy (DR) is a complex condition in diabetes that causes destruction to the sight of the eyes. It causes damage to retinal blood vessels, which are responsible for sending visual signals to the brain. DR can cause vision loss or, if not treated, even blindness. This condition occurs due to excessive sugar levels in the blood flow. This can cause the vessels to discharge fluid or blood or to become blocked, which may lead to the germination of abnormal blood vessels. These abnormal blood vessels can cause further retinal damage, causing vision loss. Changes in the retinal vasculature, such as the formation of microaneurysms, abnormal dilation or constriction, and the appearance of new blood vessels, are some of the earliest signs of diabetic retinopathy in which retinal vessels play a crucial part in early detection and monitoring. Precise segmentation of the retinal blood vessels can also help to improve the detection and diagnosis of DR by providing quantitative measurements of vessel characteristics such as branching patterns.

### Anatomy of a retina

The retina is a fine sheet of tissue that creases the rear eye and detects light to transmit visual signals to the brain. It accommodates unique cells termed photoreceptors that are delicate to light. These convert light to electrical signals, which are then refined by the other retina cells before sending these signals to the brain across the optic nerve. The retina also consists of blood vessels, nerves, and supporting cells that help to maintain its function and health. The macula at the retina’s center supervises detailed central vision. It is particularly important for driving, reading, and other activities requiring sharp vision. Diseases or conditions that affect the retina can cause vision loss or blindness. One of the common conditions that affect the retina is diabetic retinopathy.

### Vessel segmentation technique

Retinal vessel segmentation is a procedure of separating the vessels and background in an image of the retina. This process is crucial for image analysis in the medical field for diagnosing retinal diseases. The segmentation of retinal vessels involves several steps, including image preprocessing, vessel enhancement, segmentation^24^, and postprocessing. Preprocessing involves correcting for nonuniform illumination and other artifacts in the image. Vessel enhancement techniques such as vesselness filtering, Frangi filtering, or the Hessian-based filtering method enhance the contrast of the vessel structure, making it easier to distinguish it from the background. Region-growing, thresholding, graph-based or machine learning are a few segmentation algorithms that can extract the vessel structure. Postprocessing involves removing false positives or filling gaps in the segmented vessels. There are many different approaches to retinal segmentation, and the possibility of the algorithm depends on the particular application and variety of images. Some common metrics to check the accuracy of retinal vessel segmentation algorithms include specificity, sensitivity, and AUC-ROC (area under the receiver operating characteristic curve).

### Convolutional neural networks

A CNN is one of the classifications of neural networks often used for video and image processing. It is influenced by the structuring of the brain’s visual cortex that is outlined to process information with a grid-like topology, for example, images.

The main feature of a CNN is the utilization of convolutional layers that appeal to a sequence of filters to the input information. These filters are small-scale matrices of numbers that are used across the entire input image to extract features such as corners, edges, and other visual patterns. The result of each filter is termed a feature map, which is then passed through other layers of the network for further processing. Moreover, a basic CNN consists of pooling layers, which downsample the feature maps to raise the robustness of the network to small variations in the input image and to decrease the computational cost. Other layers, such as fully connected layers and activation functions, are also commonly used in CNNs. CNNs are frequently used in object detection, image segmentation, and classification. CNNs have accomplished state-of-the-art performance on many conventional datasets and are broadly used in industry and academia for various computer vision tasks.

## Methodology

### UNet

**Figure 2:**
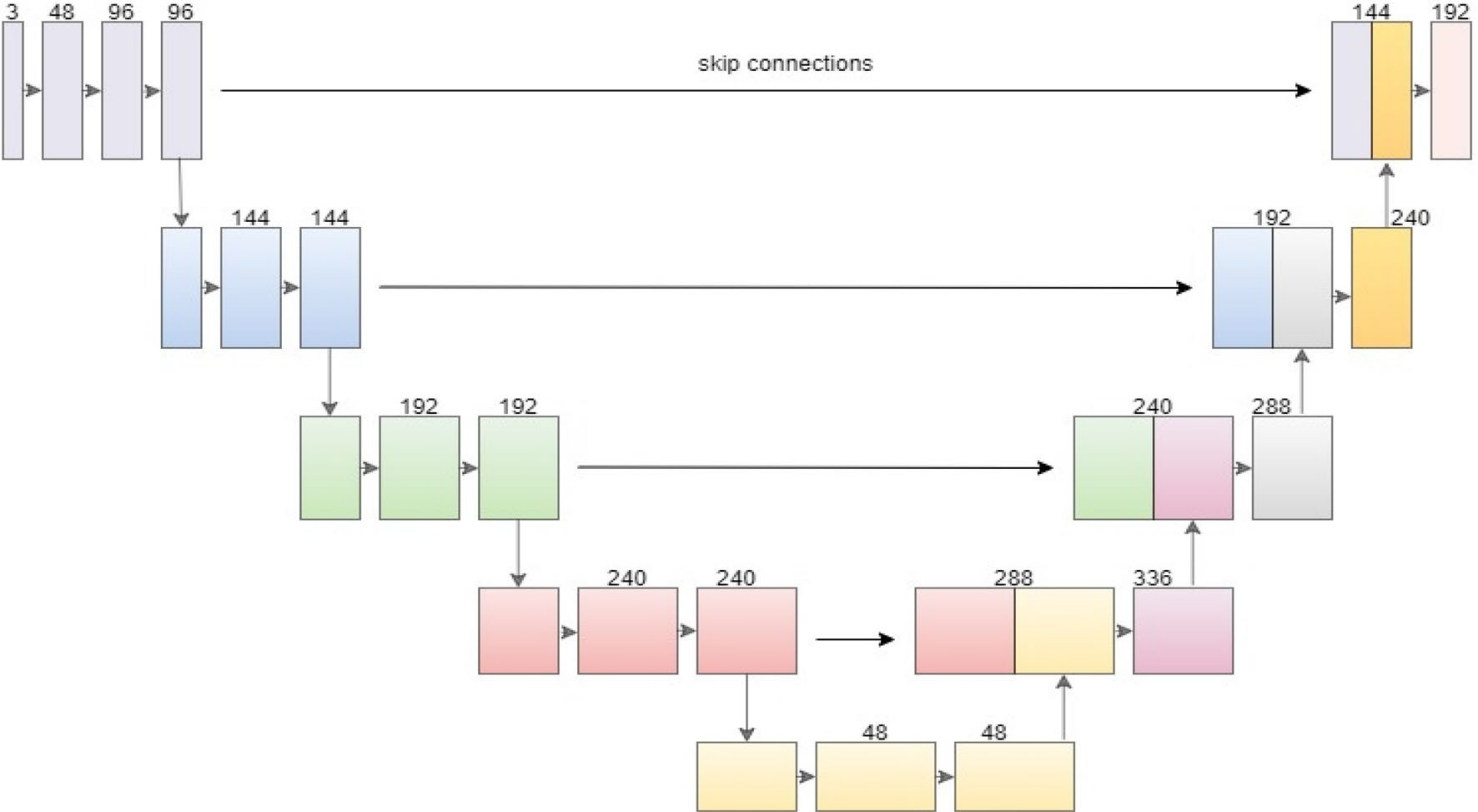
Unet architecture with right forward arrows indicating skip connections.

The proposed algorithm is established based on the UNet architecture. A UNet model is a U-shaped architecture that is particularly useful in biomedical image segmentations and is quick and accurate. According to Fig. 2, the encoder and decoder are broadly categorized parts in the U-net model. The encoder makes up the first part of the U-Net^25^ structure. Downsampling and max-pooling are carried out after a convolution block. It is a trained network representing an input image as a featured image at several levels. The decoder is located in the second half. It regulates convolution, upsampling, and concatenating pictures to generate categorized dense blocks by projecting the encoder's characteristic low-resolution feature images onto high-resolution pixel space^10^.

### Dropout block

**Figure 3:**
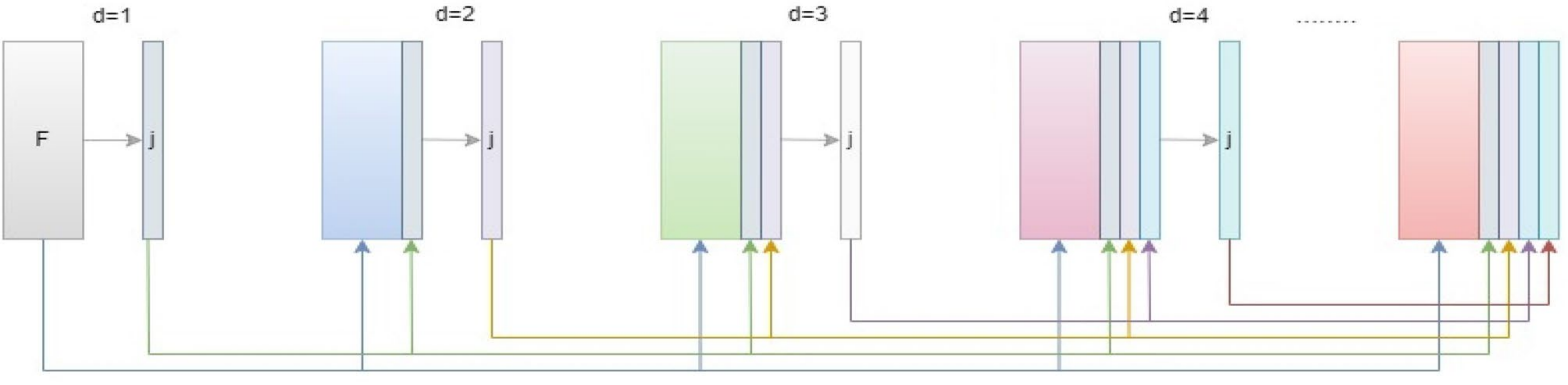
Four layers (j:12, F:48) with feature maps from the previous layer as the next layer’s input after concatenation. Colored arrows indicate concatenate connections, and black forward arrows show batch normalization, ReLU, convolution, and drop out.

The recovered features from each convolution layer may have been utilized since the fundus image numbers are constrained. Dropout blocks are added to the original UNet convolutional blocks as part of the UNet architecture to preserve the most vessel information possible and reduce overfitting, as shown in Fig. 3. A series of techniques, including batch normalization, 3 × 3 convolution, Re-LU, and dropout, are used to train the features in each layer. As a result, the output feature map j of each dth layer with (F + {d-1} x j) as an input. To lessen the model’s computational complexity, from each dense layer, only the latter-formed feature maps are assembled in the consequent layer for each four-layered dropout block, and (F + 48) feature maps are acquired as the finishing output^5^.

### Atrous channel attention

**Figure 4:**
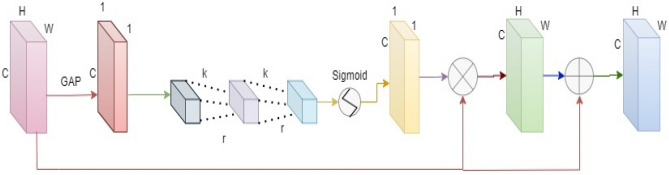
Atrous attention module with aggregating input feature maps using global average pooling generating weights after conv1D operations with kernel size k and dilation rate r.

Expanding path features are typically given equal weight in traditional segmentation networks, which cannot fully exploit appropriate data. There is a possibility that more inappropriate and irrelevant data would be preserved as a result, limiting the ability to precisely segment capillary networks. Therefore, an attention module channel called adaptive atrous is added to the model without expanding the complexity of the model to sustain each feature channel’s relevance automatically shown in Fig. 4. This module should be capable of capturing long-range dependencies that are strong in the local context of feature channels and the flexibility to understand nonlinear interactions across channels without burdening the model with additional complexity or a heavy computational load. This module includes global average pooling (GAP). GAP is utilized to generate a rectified feature characterization with each image, which is subsequently utilized to place it into one of the multiple groupings ^4^; this model can deliver realigned weight values and include two 1-D convolutions concurrently of k (kernel size) and r (dilation rate) with C as the channel dimension.

### Unfolded deep kernel estimation

**Figure 5:**
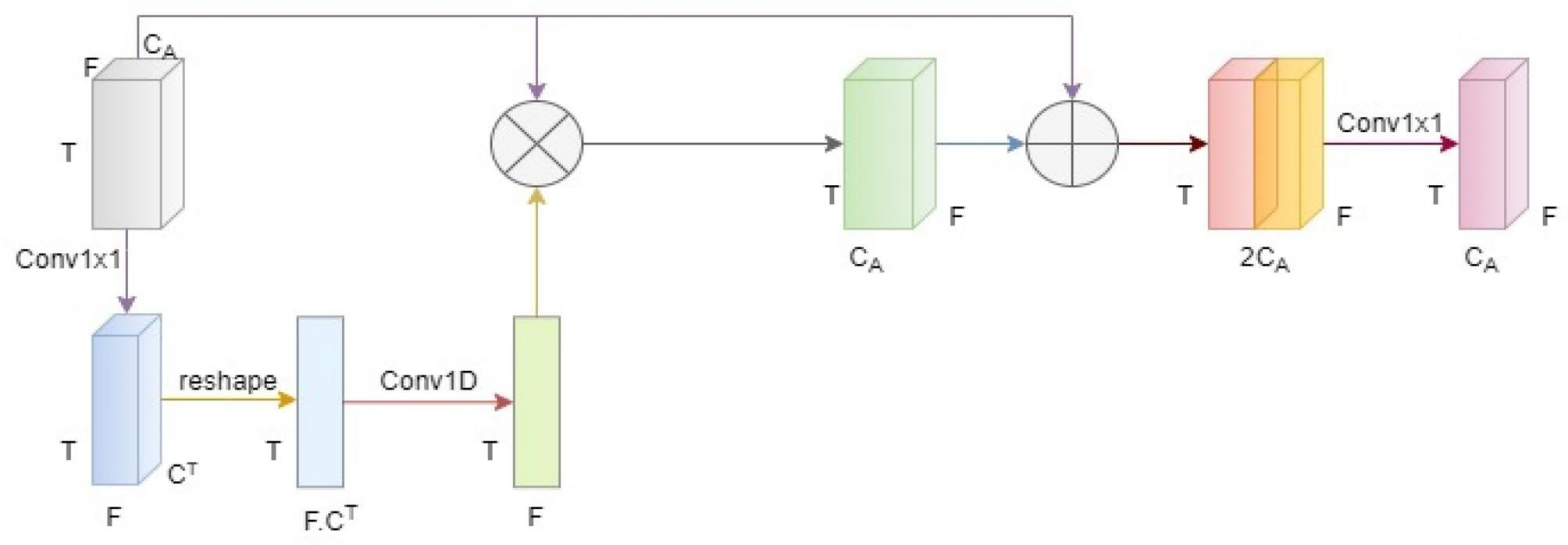
UDKE architecture with elementwise multiplication in the first half and elementwise addition in the second half follows the same process as iterations.

Unfolded deep kernel estimation (UDKE) is a technique used for image restoration that involves learning a nonlinear filter to estimate a clean image from a degraded image and kernels (filters) in a DNN, as shown in Fig. 5. It is particularly useful in cases where the degradation process is not well defined or is difficult to model explicitly, such as in blind image restoration. The UDKE algorithm defines a cost function task that estimates the difference between degraded and reconstructed clean images. This is based on unfolding iterations of an iterative algorithm and interpreting each iteration as a layer in DNN. This cost function is then optimized using a gradient descent algorithm, with the parameters of the nonlinear filter updated at each iteration^26^.

UDKE combines dense connections, knowledge distillation, and upsampling to increase the model performance. The dense connections help to improve feature reuse and preserve more information during training. Finally, upsampling is utilized to improve the feature map resolution, which can improve segmentation accuracy^27^. It can be implemented in U-Net by replacing convolution layers with UDKE layers to perform the convolution operation. The result of these convolution layers is sent into the attention block to sharpen the image by estimating the kernel.

### Multiheaded attention

**Figure 6:**
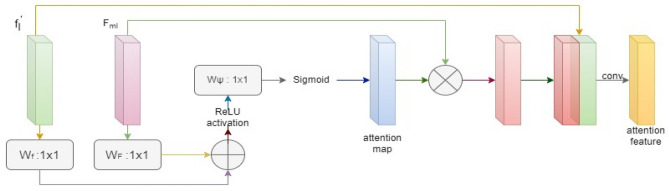
The feature attention module (FAM) obtains an attention feature map after a series of operations: ReLU and sigmoid activations and elementwise multiplication along with concatenation of both input and output feature maps followed by 3 × 3 and 1 × 1 convolution operations giving attention features.

Multiheaded characteristics are crucial to use in an implicit manner to use the matching features at each level for accurate retinal vascular segmentation. Three proceedings make up the multiheaded attention module in this case. After being concatenated, the several layered expanding path feature maps are enlarged to provide integrated multiheaded features. Second, attentional feature modules correct these characteristics in each layer. To obtain the final projected segmentation results, each attentional feature segmentation result is merged, as shown in Fig. 6. An attention gate model is employed to create an attention map rather than a sequence of convolutions. It may be trained to remove parts of the feature of the fused multiheaded block that each single-level feature such as unconnected areas that are not needed. Moreover, it can highlight desired aspects with a simpler model^3^.

### Loss function

A loss function is the summation of the branch losses connected to the auxiliary classifiers and the predicted loss of the final segmentation output^5^. It is designed to assist with the output of deep supervision and numerous branches^28^ and improve segmentation performance.

$$L=\sum_{l=1}^{L-1} {\alpha }_{l}{L}_{l}^{coarse}+\sum_{l=1}^{L-1} {\beta }_{l}{L}_{l}^{refined}+{\gamma }_{f}{L}_{f},$$

where ${L}_{l}^{coarse}$ and ${\alpha }_{l}$ represent the loss and weight prior to the *l*-th layer refinement in the segmentation map. After rectification by the FAM in the *l*-th layer, the segmentation map’s weight and loss are indicated by the notations ${L}_{l}^{refined}$ and ${\beta }_{l}$. The final output map, which is the accumulation of all the corrected forecast maps, has a loss and weight of ${L}_{f}$ and ${\gamma }_{f}$. The loss function in this instance is a cross-entropy loss, in which a metric is utilized to calculate the conduct of an ML (machine learning) model measured in range (0, 1), with 0 and 1 denoting a perfect model and an inaccurate model, respectively. Finally, the parameters are optimized using the Adam optimizer ^29^ by fully decreasing the loss function ($L$).

### Architecture overview

**Figure 7:**
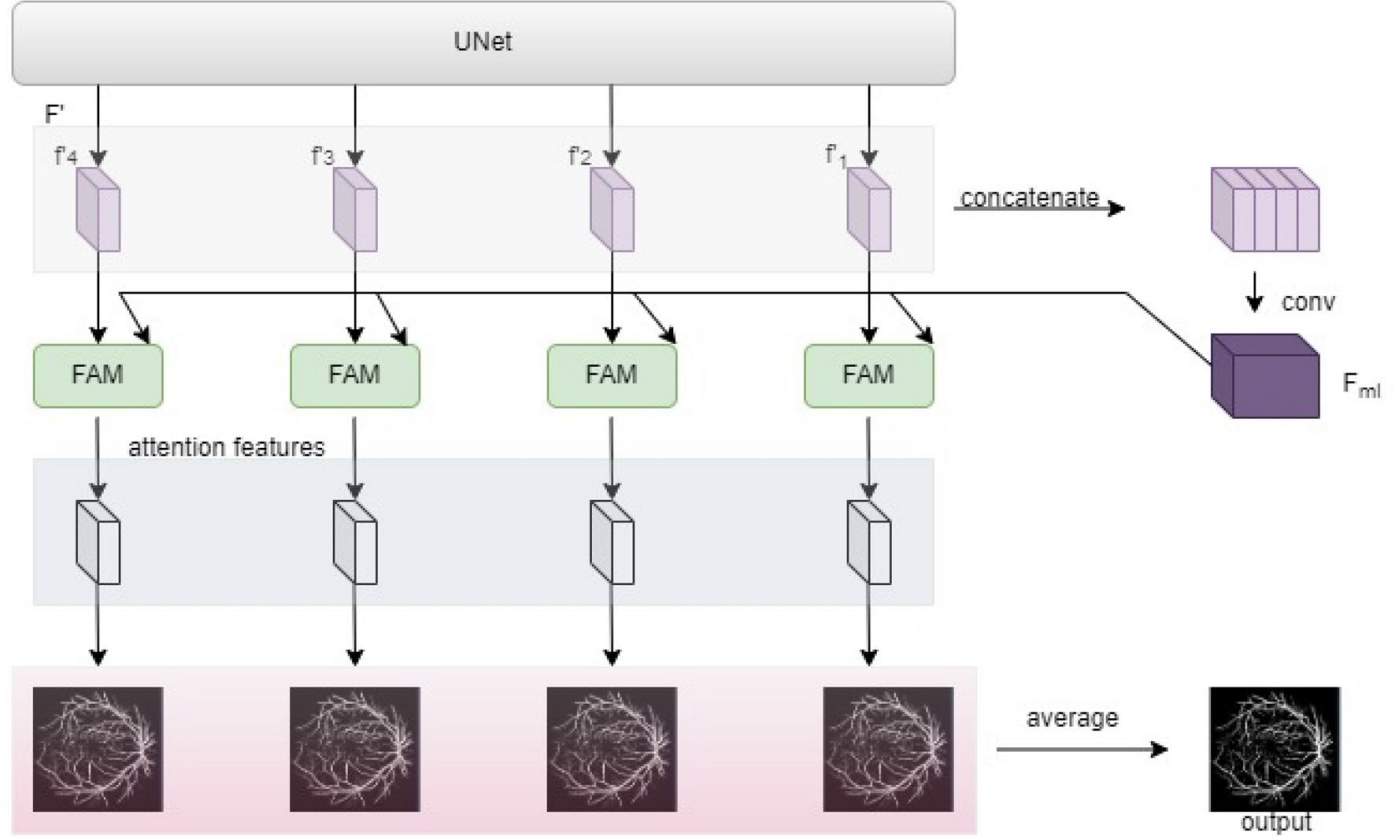
Architecture showing output obtained from the average of all refined images from attention features that are extracted from the feature attention module after concatenation and convolution of input feature maps obtained as the output of UNet module that performs dropout, udke, and atrous attention module to extract features from preprocessed input retinal image.

The architecture has two main parts: a multilevel feature extraction algorithm and a multiheaded attention algorithm. The first module comprises various convolutional layers that extract features of distinct levels of abstraction from the input image. The resulting feature of each convolutional layer is fed to a max-pooling to deduct the spatial dimension of each feature map. Encoding–decoding paths make up the entire UNet architecture for vessel segmentation. The dropout block and the Atrous Channel Attention Block are included in the encoding pathway, followed by downsampling. The dropout block is also used in decoding, and an upsampling convolution transpose layer is displayed. Further, these feature maps are put into an udke block to obtain a high-resolution image. Skip connections between encode and decode pathways are performed to link each feature. Bilinear interpolation is utilized to extend the decoding path’s feature maps, and these features are then fed as input of the multiheaded attention module as shown in Fig. 7.

The attention network comprises several modules operating on the feature extraction network output. Each attention module includes two parts: query and key value branch. The query branch generates a query vector that encodes the feature map spatial information. The key value branch generates vectors of value and key that encode the channel-wise data of feature maps. The query vector is then used to attend to the key-value pairs, generating an attention map pointing out the relevant segmentation features. The result of the attention network is then passed to a convolutional decoder network that generates the final vessel segmentation mask. The decoder network has several upsampling layers that increase the feature map spatial resolution and several convolutional layers that merge the different levels of abstraction feature maps. The proposed model is trained using a binary cross-entropy loss function and an Adam optimizer. The performance of this model is estimated on three convenient datasets, CHASE_DB1, DRIVE, and STARE showing effective production in accuracy.

## Implementation

### Preprocessing

Three databases are utilized for evaluation: DRIVE, CHASE_DB1, and STARE. As the CHASE_DB1 and STARE datasets lack binary FOV masks, they must be produced manually, similar to^15^. The hue and saturation of a single retinal color picture vary greatly. Each elementary picture should be transformed into each intensity image, which should then be further normalized to have unit variance and zero means. The normalized strengths are then scaled to the range (0, 255).

**Figure 8:**
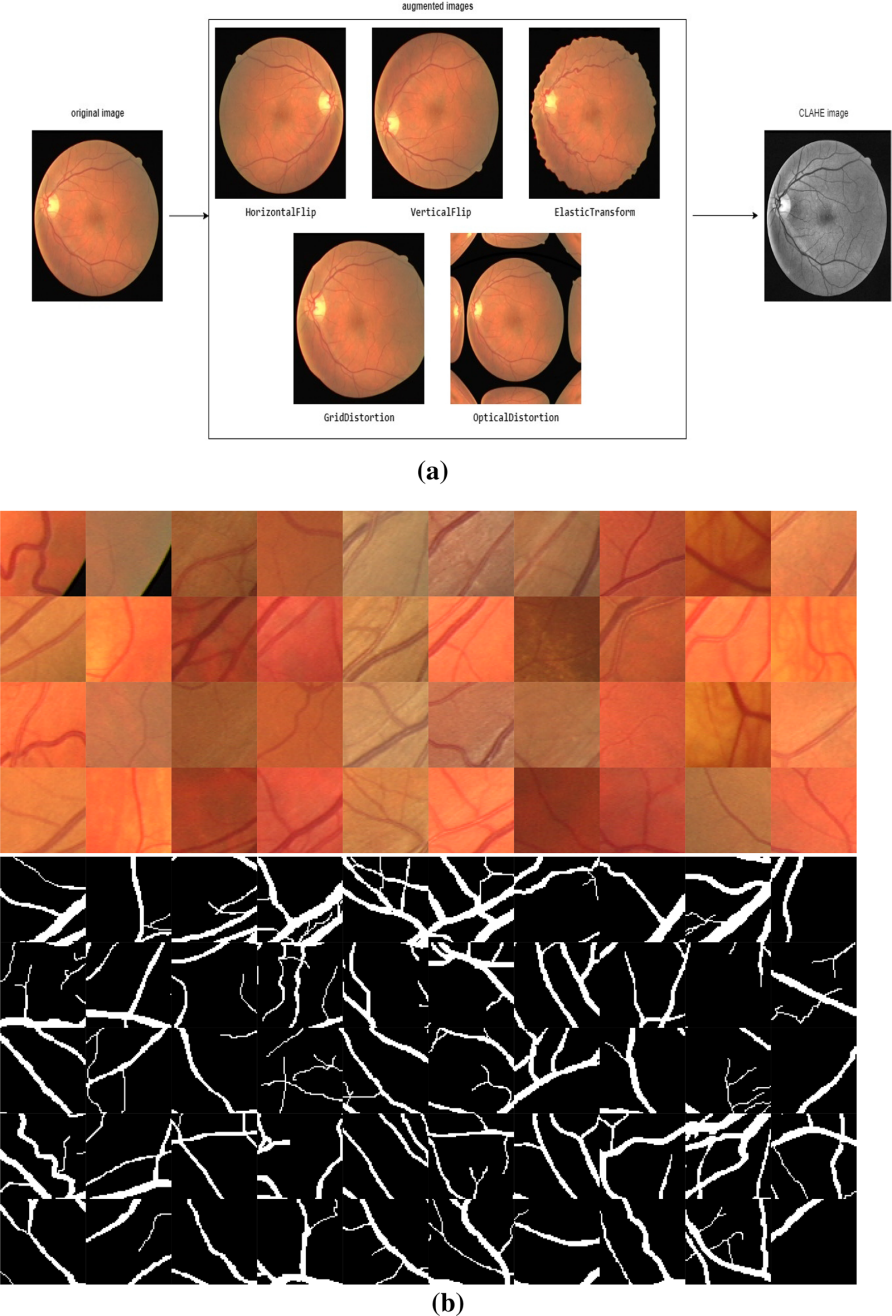
(**a**) Data preprocessing using data augmentation, CLAHE, and gamma correction. (**b**) Divided image patches after preprocessing with a size of 256 × 256 for training.

In human vision, a gamma adjustment algorithm is used to acquire the processed pictures*.* Data augmentation and CLAHE are commonly used preprocessing techniques for retinal vessel segmentation. They can improve the quantity and quality of the available information, ultimately increasing the segmentation model performance. Data augmentation includes producing new images by appertaining different transformations to the initial database. This can include flipping, rotating, zooming, and shifting the images, among other techniques shown in Fig. 8a. By doing so, the augmented data can increase the distinctiveness of the training data. They may help prevent overfitting, which can occur when a model is too specific to the original information and does not establish well to new information.

The CLAHE^30^ technique is obtained to amplify the contrast of the retinal image. It controls the severe noise amplification in the nearby regions to solve the poor contrast of low intensities. CLAHE is utilized to strengthen the contrast of retinal images. This CLAHE algorithm can increase the contrast of the retinal image while maintaining the overall brightness and color balance, which can help differentiate the retinal vessels in the background. This can enhance the accuracy of the segmentation model, especially in cases where the contrast between the background and vessels is low.

Further, to increase the dataset size, We utilized three datasets: DRIVE, STARE, and CHASE_DB1. The DRIVE dataset comprises 20 images, each with a size of 565 × 584. The STARE dataset includes 10 images with a size of 700 × 605, while CHASE_DB1 contains 14 images with a size of 999 × 960. We extracted patches of size 256 × 256 and applied five different augmentation techniques. Overall, we used 1200 image patches of size 256 × 256 in our work, as shown in Fig. 8b.

### Training and testing

To control the overfitting problem, data augmentation, and patch-based segmentation are employed while training the model. Modifications such as flipping and rotating the image are employed in the training set on each picture to obtain enhanced images.

**Figure 9:**
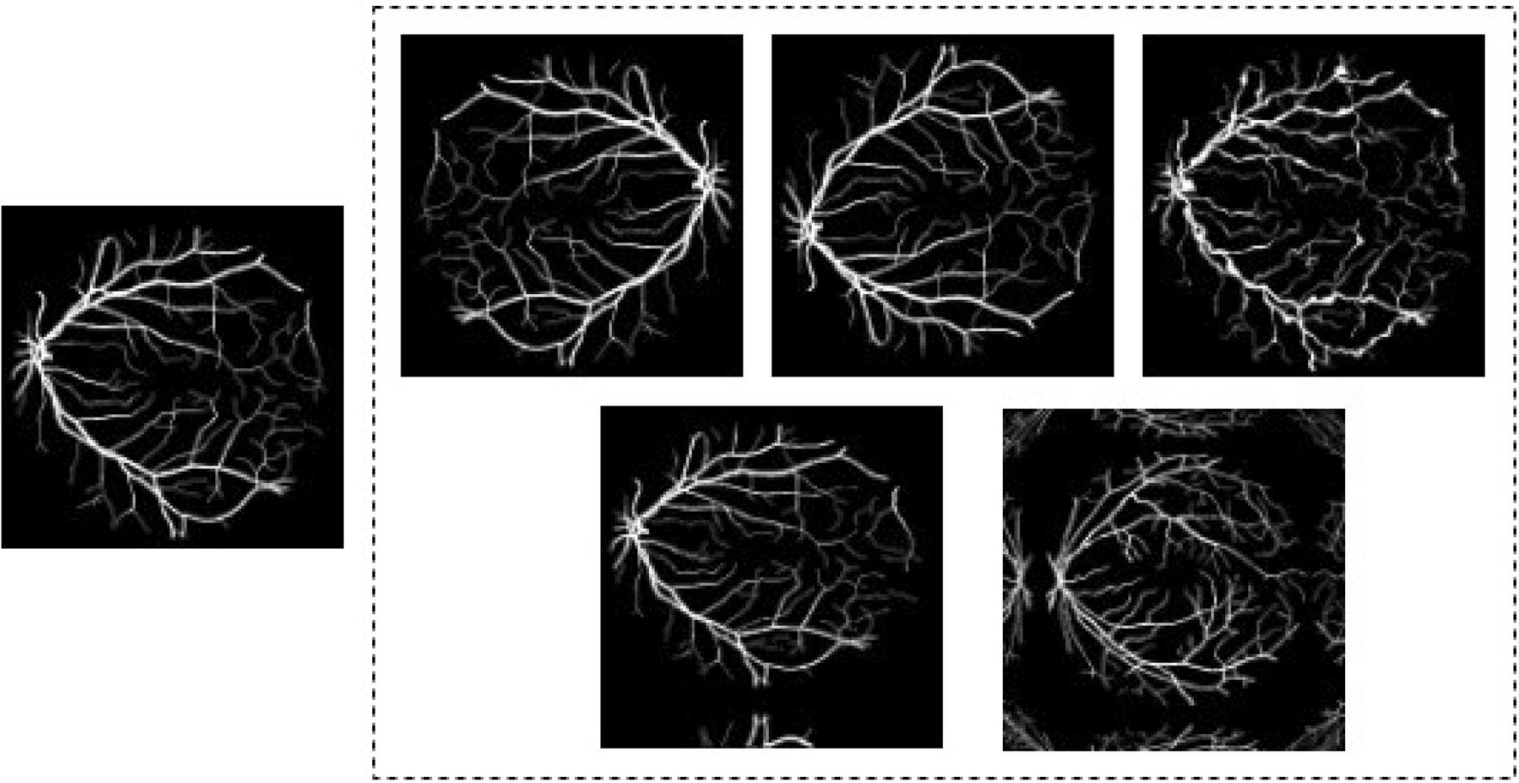
Segmented images used in training the model.

A small batch of images of the model for training uses the size-4 Adam optimizer^29^. Each test picture is first preprocessed with a size of 256 overlapping patches removed with a stride of 20. They are then supplied with a segmentation model that has been fully trained, and the maps of segmented vessels are gathered as a series of cropping processes and are further averaged in overlapping areas. To establish whether there is a specific difference between the suggested approach and the two UNet ROC correlation curves, Wilcoxon’s rank sum test^31^ is used under supervision. Figure 9 shows the ground truth images used to train the model.

### Evaluation metrics

In vessel segmentation, various evaluation metrics can be used to estimate the segmentation model performance, such as accuracy, F1 score, Jaccard score, recall score, and precision. The assessment metrics are built on the number of TPs and TNs (true-positives and true-negatives) and FPs and FNs (false-positives and false-negatives), which are derived from a comparison of the predicted binary vessel map to the ground truth binary vessel map to assess the segmentation findings to solve the issue of binary segmentation of retinal vessels. TPs are the number of pixels correctly classified as vessels in the two maps of both ground truth and predicted image. TNs are the number of pixels correctly distinguished as non-vessels in both maps. FPs are the number of pixels distinguished as vessels in the predicted map but are not vessels in the ground truth map. FNs are the number of pixels classified as vessels in the ground truth map but are not distinguished as vessels in the predicted map.

Accuracy is the proportion of correctly distinguished pixels in the predicted binary map computed as [(TP + TN)/(TP + TN + FP + FN)]. Precision is the proportion of predicted vessels correctly recognized by this model computed as [TP/(TP + FP)]. Sensitivity, also known as recall, is the fraction of true vessels correctly recognized by this model computed as [TP/(TP + FN)]. F1-score is the harmonic mean of sensitivity and precision, or it is a stabilized evaluation that takes both FPs and FNs into account and is computed as [(2 × {precision × recall})/(precision + recall)]. Jaccard score, also known as IoU (intersection over union), estimates the underlying ground truth and predicted binary maps and is calculated as [TP/(TP + FP + FN)]. These evaluation metrics are utilized to compare the performance rate of different vessel segmentation models and optimize the segmentation algorithm's parameters for better performance.

## Results

### Performance metrics results

The network performance on the performance metrics is potentially improved, especially on spatial accuracy and detail preservation metrics, such as accuracy, IoU, and F1 score. The outputs of the proposed method have shown that it accomplished high performance on datasets, outperforming several state-of-the-art methods. This proposed model has also accomplished an accuracy of more than 96.1%, an F1 score of more than 72.2%, a Jaccard score of more than 55.5%, a recall score of more than 75.9%, and a precision score of more than 61.1%, as mentioned in Table 1.

**Table 1:** Performance metrics for three datasets.

Database	Accuracy	Precision	F1 score	Jaccard score	Sensitivity
DRIVE	0.9612	0.6112	0.8295	0.5551	0.7657
STARE	0.9671	0.7264	0.7228	0.5945	0.7591
CHASE_DB1	0.9629	0.7156	0.7493	0.6084	0.8302

### Comparing training and validation metrics

Comparing the validation and training metrics can help to understand the model performance on unrevealed information. If the validation metrics are constantly less than the training metrics, it might specify that the algorithm is overfitting compared to the training data. In contrast, if the validation metrics are consistently higher than the training metrics, it might indicate that the model is underfitting and needs more training data.

Validation loss evaluates how well an algorithm performs on a set of validations during training. It represents the differences between predicted and ground truth image segmentation in the validations. The lower the validation loss, the better the model performance on a validation set. Validation accuracy measures the model’s prediction of vessel segmentation in a validation set. It represents the ratio of pixels correctly distinguished from the total validation set pixel number. The more the validation accuracy, the higher the model’s performance on the validation set. The validation IoU coefficient measures how well-predicted segmentation overlaps with a ground truth segmentation in a validation set. The higher the validation IoU coefficient is, the better the model performance on a validation set.

### Comparison with other state-of-the-art methods

**Figure 10:**
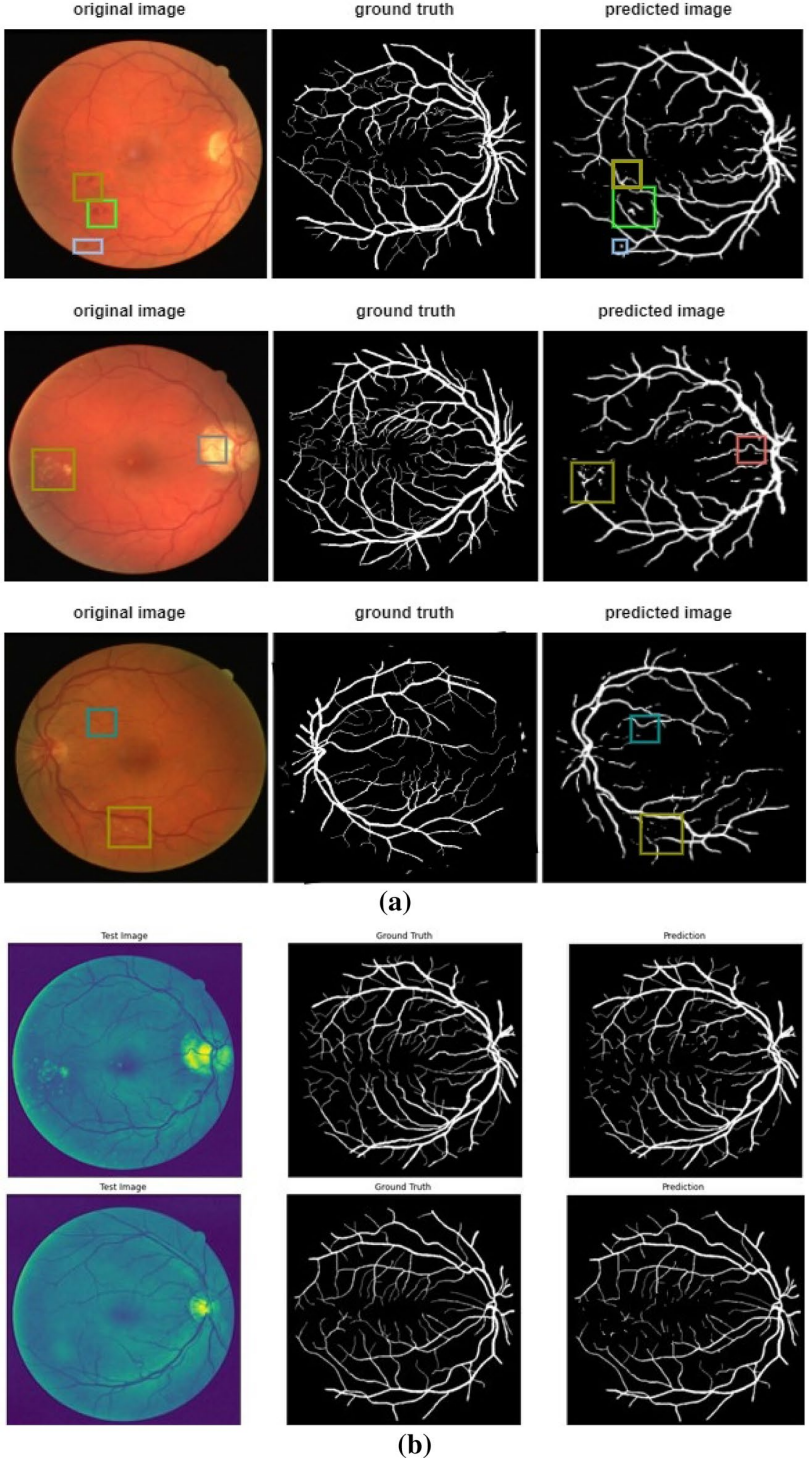
(**a**) Predicted vessel map comparison with Groundtruth. (**b**) Predicted vessel map comparison with Groundtruth when tested in a network with green channel image.

The proposed algorithm is compared to recent state-of-the-art models in retinal vessel segmentation. Specifically, UNet^9, 16–18,32^, filters or layers^19, 20^, wavelet transform^21^, attention^22^, and modality^23^ are related methods. To evaluate the different method performances, metrics including F1-score, accuracy, and sensitivity are compared. The results show the model performance compared to recent state-of-the-art methods in achieving accuracies of 0.9612 and 0.9671 for the DRIVE and STARE databases, respectively, which are significantly higher than those of other methods. Additionally, our proposed model achieved a sensitivity of 0.8302 in CHASE_DB1. The F1 score for the DRIVE dataset with this proposed method is 0.8295, higher than the mentioned models. Segmented results of the proposed model are shown in Fig. 10a. Therefore, by adding udke and the attention module, vessel segmentation accuracy has increased compared to the other state-of-the-art models mentioned in Table 2. Table 3 shows the confusion matrix and the accuracy for the single image. A confusion matrix is calculated between the model’s predicted vessel map and the corresponding ground truth.

**Table 2:** Comparison with the recent state-of-the-art methods.

	DRIVE			STARE			CHASE_DB1		
	Acc	Sen	F1	Acc	Sen	F1	Acc	Sen	F1
BI-WT and RF^21^	0.9466	0.7953	0.7861	0.9547	0.7882	0.7815	0.9502	0.7644	0.7581
Cross-modality^23^	0.9527	0.7569	–	0.9628	0.7726	–	0.9581	0.7507	–
D-UNet^16^	0.9566	0.7963	0.8237	0.9641	0.7595	**0.8143**	0.9610	0.8155	0.7883
R2U-Net^17^	0.9556	0.7799	0.8171	–	–	–	0.9634	0.7756	0.7928
Visual attention^22^	0.9589	**0.8644**	0.7607	0.9502	**0.8325**	0.7698	0.9474	0.8297	0.7189
Res-UNet^18^	0.9553	0.7726	0.8149	–	–	–	0.7800	0.7726	**0.9553**
3-stage DL^19^	0.9538	0.7631	–	0.9638	0.7735	–	0.9607	0.7640	–
DE-UNet^9^	0.9567	0.7940	0.8270	–	–	–	0.9661	0.8074	0.8037
Trainable COSFIRE filter^20^	0.9442	0.7655	–	0.9497	0.7716	–	0.9387	0.7585	–
Proposed method	**0.9612**	0.7657	**0.8295**	**0.9671**	0.7591	0.7228	0.9629	0.8302	0.7493

(Bold values indicate the response achieved by a particular method compared to other mentioned algorithms).

**Table 3:** Confusion matrix of the predicted vessel map and the ground.

Confusion matrix of output images [(TN) (FP) (FN) (TP)]	
[[58445 3603] [3453 798]]	This is the confusion matrix for the image (row 1) in Fig. 11a. The proposed model accurately predicted vessel and non-vessel pixels in the fundus image with an overall accuracy of 89.4%
[[58342 3263] [3187 744]]	This is the confusion matrix for the image (row 2) in Fig. 11a. The proposed model accurately predicted vessel and non-vessel pixels in the fundus image with an overall accuracy of 90.16%
[[56742 3929] [3612 1253]]	This is the confusion matrix for the image (row 3) in Fig. 11a. The proposed model accurately predicted vessel and non-vessel pixels in the fundus image with an overall accuracy of 88.41%
[[53648 1816] [2457 7615]]	This is the confusion matrix for the image (row 1) in Fig. 11b. The proposed model accurately predicted vessel and non-vessel pixels in the fundus image with an overall accuracy of 74.14%
[[57598 2177] [2941 2820]]	This is the confusion matrix for the image (row 2) in Fig. 11b. The proposed model accurately predicted vessel and non-vessel pixels in the fundus image with an overall accuracy of 92.30%

**Figure 11:**
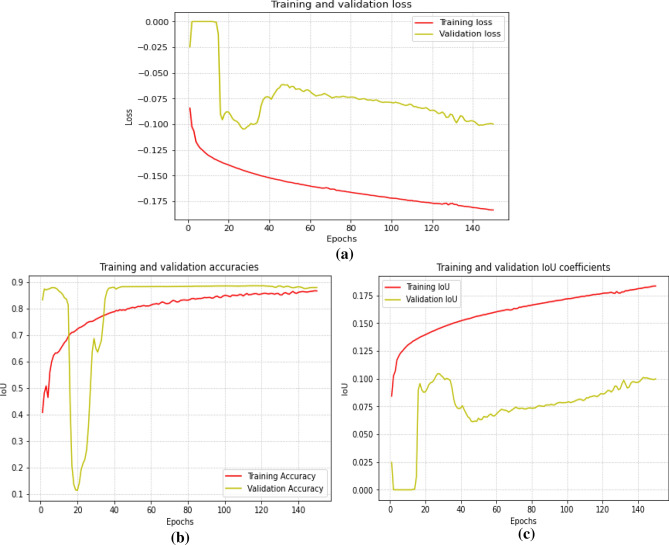
Graphs showing (**a**) validation loss, (**b**) validation accuracy, and (**c**) validation IoU coefficients.

## Discussion

About correctly recognizing and segmenting vessels in retinal pictures, the UDKE multiheaded attention network for retinal vascular segmentation has demonstrated encouraging results. The suggested method focuses on particular image areas and extracts pertinent features for segmentation using global and local attention mechanisms. The results have shown better improvement in achieving an accuracy of 0.9612, 0.9671, and 0.9629 in the DRIVE, STARE, and CHASE_DB1 databases, respectively, which is significantly a good percentage compared to the other methods. Our proposed model also achieved 0.7657, 0.7591, and 0.8302 sensitivity and F1 scores of 0.82.95, 0.7228, and 0.7493 for the DRIVE, STARE, and CHASE_DB1 datasets, proving that the model has achieved better results in accuracy in comparison with the other state-of-art models.

Moreover, adding more reliable and diversified training data is a possible future enhancement. The network has only been trained on a small sample of pictures, which may not accurately represent the range of variability seen in retinal scans. A larger and more varied training dataset might aid the network’s ability to generalize to fresh pictures, enhancing its performance on previously unexplored material. The udke multiheaded attention network might also be improved by using different modalities in addition to conventional grayscale pictures. For instance, adding channels such as fluorescein angiography or optical coherence tomography might give the network more context and information to consider when segmenting veins. Therefore, retinal vascular segmentation using the multilevel attention network can significantly increase the precision and effectiveness of vessel analysis of the retina in the future. Figure 11 shows the proposed model’s training and validation performance; a sudden decrease in the validation accuracy and IoU represents the lack of ground truth training dataset. In the future, we can work on possible ways to utilize the limited training dataset to increase the model’s generalization ability.

## Conclusion

The framework is used to segment retinal vessels. The dropout block is used instead of an initial convolution block to reduce overfitting and preserve the most vessel information possible among convolution layers. The atrous channel attention module is automatically inserted to rank the relevance of each feature channel in the encoding pipeline. The UDKE block enhances the image's resolution through iterations by passing feature maps into a dense UDKE module by calculating the blur kernel to obtain a high resolution from low-resolution feature maps. The multiheaded attention module is introduced to fully utilize multilayer decoding route features and combine them to further improve features at each level.

The result shows that this proposed network performs well compared to all of the state-of-the-art models mentioned in achieving accuracy in the DRIVE and STARE databases of 96.12% and 96.71%, respectively, significantly higher than the scores achieved by the other methods. The method proposed in Ref.^9^ performed better in acquiring an accuracy of 96.61% for the CHASE_DB1 dataset. Regarding sensitivity, the method proposed in Ref.^22^ acquired 86.44% for DRIVE and 83.25% for the STARE dataset, and our proposed method achieved a sensitivity of 83.02% in CHASE_DB1 compared to other methods. F1 scores for STARE and CHASE_DB1 are higher for the methods used in Ref.^16^ and^18^ at 81.43% and 95.53%, respectively, whereas the F1 score for the DRIVE dataset with this proposed model is 82.95%, which is higher compared to the mentioned models. The results of this approach, which is based on a multiheaded attention network that combines the UNet architecture with a hierarchical attention mechanism, illustrate that this model outperforms several other state-of-the-art methods on the datasets across all evaluation metrics. Specifically, this method attains the highest F1 score for the DRIVE dataset, high accuracy for both the STARE and DRIVE datasets and high sensitivity for the CHASE_DB1 dataset, indicating its superior performance in accurately segmenting retinal blood vessels. The approach is capable of adaptation and works well in challenging instances. As a prospect, one can further develop a more detailed model that aids in identifying vessels with more specificity and enlarging the vascular structure’s connectedness. Further, improving deep learning models' interpretability is crucial for gaining insights into the regions of interest detected by the model. To achieve this goal, visualization methods can be utilized. These techniques can assist in providing a clear understanding of the inner workings of the model, enabling better decision-making and more effective problem-solving.

## Data Availability

The authors have used publically available data in this manuscript. The dataset link is mentioned in the reference list.
